# Unsuppressed viral load after intensive adherence counselling in rural eastern Uganda; a case of Kamuli district, Uganda

**DOI:** 10.1186/s12889-021-12366-4

**Published:** 2021-12-18

**Authors:** Geoffrey Ndikabona, John Bosco Alege, Nicholas Sebuliba Kirirabwa, Derrick Kimuli

**Affiliations:** 1 Institute of Public Health, Clarke International University, P.O. Box 7782, Uganda Kampala,; 2 Center for Human Services, University Research Co., LLC, Kampala Uganda P.O. Box 28745, Kampala,; 3 Directorate of Socio-Economic Surveys, Uganda Bureau of Statistics, Kampala, Uganda, P.O. Box 7186, Kampala, Uganda

**Keywords:** Viral suppression, Intensive adherence counselling, Antiretroviral therapy, Kamuli district

## Abstract

**Background:**

The East Central (EC) region of Uganda has the least viral suppression rate despite having a relatively low prevalence of human immunodeficiency virus (HIV). Although the viral suppression rate in Kamuli district is higher than that observed in some of the districts in the region, the district has one of the largest populations of people living with HIV (PLHIV). We sought to examine the factors associated with viral suppression after the provision of intensive adherence counselling (IAC) among PLHIV in the district.

**Methods:**

We reviewed records of PLHIV and used them to construct a retrospective cohort of patients that started and completed IAC during January – December 2019 at three high volume HIV treatment facilities in Kamuli district. We also conducted key informant interviews of focal persons at the study sites. We summarized the data descriptively, tested differences in the outcome (viral suppression after IAC) using chi-square and t-tests, and established independently associated factors using log-binomial regression analysis with robust standard errors at 5% statistical significance level using STATA version 15.

**Results:**

We reviewed 283 records of PLHIV. The mean age of the participants was 35.06 (SD 18.36) years. The majority of the participants were female (56.89%, 161/283). The viral suppression rate after IAC was 74.20% (210/283). The most frequent barriers to ART adherence reported were forgetfulness 166 (58.66%) and changes in the daily routine 130 (45.94). At multivariable analysis, participants that had a pre-IAC viral load that was greater than 2000 copies/ml [adjusted Prevalence Risk Ratio (aPRR)= 0.81 (0.70 - 0.93), p=0.002] and those that had a previous history of viral load un-suppression [aPRR= 0.79 (0.66 - 0.94), p=0.007] were less likely to achieve a suppressed viral load after IAC. ART drug shortages were rare, ART clinic working hours were convenient for clients and ART clinic staff received training in IAC.

**Conclusion:**

Despite the consistency in drug availability, counselling training, flexible and frequent ART clinic days, the viral suppression rate after IAC did not meet recommended targets. A high viral load before IAC and a viral rebound were independently associated with having an unsuppressed viral load after IAC. IAC alone may not be enough to achieve viral suppression among PLHIV. To improve viral suppression rates after IAC, other complementary services should be paired with IAC.

## Background

Over 38 million people globally are living with the human immunodeficiency virus (HIV) [1], and more than half (53%) of whom are estimated to have an unsuppressed viral load [2]. The globally consolidated efforts aim to ensure by that by 2030, 95% of all people living with HIV (PLHIV) that are receiving antiretroviral therapy (ART) have a suppressed viral load [2]. However, the number of PLHIV who have initiated treatment but are not virally suppressed (viral suppression gap) increased from 18% in 2015 to 21% in 2017 [3]. Achieving sufficient viral suppression among PLHIV remains a challenge in many countries [1, 4–7] leading to increased transmission of HIV [8–11]. The World Health Organization (WHO) through the “Consolidated guidelines on the use of antiretroviral drugs for treating and preventing HIV infection” recommends the use of intensive adherence counselling (IAC) as the best strategy to achieve viral suppression [12].

IAC is a continual and repeated process initiated by health providers to support patients to identify possible barriers and resolutions to ART adherence. IAC is provided through various individualized processes including education sessions, counselling, and assessments [5, 13]. By providing IAC, health workers support clients to improve ART adherence and consequently, viral suppression. While there was a near-universal adoption of these guidelines as national country policies [14], the quality of IAC received by patients was unclear and could partly contribute to the increasing viral suppression gap [3]. PLHIV with unsuppressed viral loads are at risk of a severely weakened immune system and susceptible to opportunistic infections, which may result in a higher mortality [15, 16]. In addition, a high viral load increases the chance of transmission of HIV, increasing the incidence of infection, the burden on health resources, among other concerns [11, 17].

In 2016, the Ministry of Health Uganda (MoH) adopted the WHO guidelines requiring all PLHIV with a viral load above 1000 copies to receive IAC [12, 18–20]. According to the Joint United Nations Programme on HIV/AIDS (UNAIDS) in Uganda, it was estimated that there are more than 1.5 million PLHIV in the country and only 64% of those receiving ART were virally suppressed [10]. In the East-Central (EC) region of Uganda, Kamuli district is one of the districts that host the majority of PLHIV, however, only about 82.4% of PLHIV on ART are virally suppressed [12]. The region and district fall short on performance levels for some of the most critical health and wellbeing indicators regarding poverty, child health and sexually transmitted diseases [21]. Therefore, we studied the viral suppression and factors associated with viral suppression after IAC as per the MoH/WHO guidelines [12, 18–20] during routine program implementation in Kamuli district. The findings are critical for improving the implementation of strategies aimed at improving viral suppression in the district and similar settings.

## Methods

### Study design and setting

We conducted both a retrospective and a cross-sectional study but used complementary discussed the findings. For the retrospective aspect, we reviewed health facility records of a cohort of patients that started and completed on IAC between the periods of January 2019 – December 2019. This cohort was chosen because it was the most recent cohort for which all patients had completed IAC by the study period time. We collected data on IAC, viral suppression, socio-demographic, personal and other clinical characteristics through data triangulation from MoH client files, ART Registers, Counselling forms, and the Non-suppressed viral load register. The data were collected using a pretested quantitative questionnaire.

For the cross-sectional aspect, ART clinic focal persons for the three sites were chosen as key informants for interviews. These were chosen because they provided the most holistic information about viral suppression and IAC at the study sites. The ART clinic focal persons supported responses to questions on the health-related factors during the period under study for the clients. These data were used to provide narratives and explanations related to practices and experiences at the health facilities during the study period. The data were collected using a key informant interview guide.

### Study population

The primary study population was all available records of PLHIV that were virally unsuppressed who started, and completed IAC between January 2019 – December 2019 at Kamuli General Hospital, Namwendwa Heath Centre (HC) IV, and Nankandulo HC IV in Kamuli district. These facilities were chosen because they attend to the highest proportion of HIV patients in the district. Only records of PLHIV who had started and completed IAC were included because they had a viral load test result available at the end of IAC. PLHIV that didn’t complete IAC did not have a viral load test after IAC, albeit, the MoH guidelines stipulate that the assessment of viral suppression after IAC should be done after successful completion of IAC [20]. An ART Clinic In-Charge at each of these ART clinics was chosen as the second study population for the qualitative interview.

### Eligibility criteria

#### Inclusion criteria

All records of PLHIV who were unsuppressed started and completed IAC between January 2019 – December 2019 and all ART Clinic In-charges working at Kamuli Hospital, Namwendwa HC IV, and Nankandulo HC IV were included in the study.

#### Exclusion criteria

The study excluded the records of the following participants, virally unsuppressed PLHIV that were not started on IAC, started but not during the period January 2019 – December 2019, started on but didn’t complete IAC during January 2019 – December 2019, health workers not working at the ART clinic at Kamuli General Hospital, Namwedwa HC IV, and Nankandulo HC IV and all PLHIV referred from any other health centres in the region.

#### Research variables

The outcome of the study was viral suppression after IAC. It was categorized into suppressed (Yes/ less than 1000 copies/ml of blood) and unsuppressed (No / more than 1000 copies /ml of blood). Viral suppression for this study was defined as the suppression of viral load to less than 1000 copies/ml of blood after a repeat viral load at the end of 3 consecutive IACs that are one month apart with good adherence. The independent variables included the patient’s socio-demographic, personal and clinical characteristics.

#### Sample size estimation

The study was a retrospective review of 283 records of the non-suppressed viral load register ART registers, MOH client files and counselling forms using a census of all records of HIV+ of unsuppressed clients who started and completed IAC between January 2019 – December 2019 at Kamuli General Hospital, Namwendwa HC IV and Nankandulo HC IV. A census was conducted because the virally unsuppressed population in Kamuli district is relatively small and well-defined. This eliminated potential bias that occurred through using a sampling technique and it was the best use of the limited time and resources available for this study. The study site facilities hosted the majority of PLHIV who are unsuppressed within the districts offering a more than fair chance of representation of the findings. The study period offered the best chance of gathering the most recent and complete information available for the study objectives, and all patients within the chosen period had completed IAC period by the time of the study. Key informant interviews were conducted among ART Clinic In-Charges who were purposely chosen because of their unique experience and understanding of aspects regarding HIV care and IAC at their health facilities. They were HIV Focal Persons of the clinics so their selection for the study was strategic and supported explanatory aspects of the findings.

#### Sampling procedures

A complete enumeration of records of all HIV+ unsuppressed clients who started and completed IAC between January 2019 – December 2019 at Kamuli hospital, Nankadulo HC IV, and Namwedwa HC IV was be made. For each of the records listed, data was collected on the objectives from MOH Client files, ART Registers, Counselling forms, and the Non-Suppressed viral load register into a pre-designed questionnaire. Each ART In-Charges at this health was purposively selected for a key informant interview. One key informant interview per health facility was conducted.

#### Study procedures

The study primarily involved the collection of quantitative data from MOH client files, ART registers, counselling forms, and the non-suppressed viral load register using a pre-designed questionnaire. The collection of qualitative data for the study was done by conducting a one-on-one in-depth interview with an ART Clinic In-Charge of the selected health facilities in the district using a predesigned questionnaire.

#### Data management and analysis

The data was double-blind captured in KoBo Collect and exported into Microsoft Excel, cleaned, and analyzed using STATA 15, for analysis. Depiction of the discoveries was made utilizing relevant measurements. Summary data were aggregated and presented using frequencies and rates for variables that are grouped (categorical), however, for continuous variables such as age, averages were presented with standard deviations (SD). Pearson’s chi-squared and Fisher’s tests were used to confirm or nullify the existence of a relationship between the outcome variable (dependent) and the independent variables for the study. A log-binomial regression analysis with robust standard errors at a 5% statistical significance level was used to assess the association, risk ratios and corresponding confidence intervals (CI) were reported. Recorded interviews were transcribed verbatim transcriptionists, coded, harmonized, themed, and verified. The researcher themed transcriptions into major and common discussion areas for summative narrative. Under the broad theme Influencers of viral suppression, three subthemes were established, (1) Drug availability, (2) Staffing adequacy and (3) Patient follow up procedures. Some highlights/excerpts from the analysis were quoted verbatim.

## Results

### Study profile

Between January – December 2019, a total of 354 PLHIV were reported to be virally unsuppressed at the three health facilities of whom 316 were enrolled on IAC. We excluded 71 records for reasons: 38 were not enrolled on IAC, 24 did not complete IAC and 9 were incomplete. Overall, 283 records were included in the study.

### Description of the study population

**Fig. 1:**
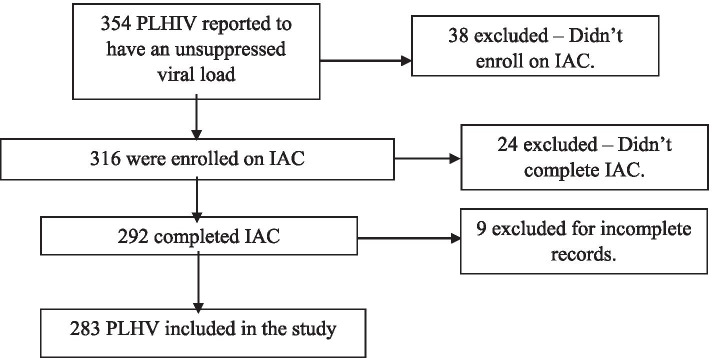
Study profile

Figure 1 above shows the characteristics of the study participants. The records of 283 patients who had started and completed IAC during January – December 2019 were reviewed.

### Socio-demographic characteristics

The mean age of the participants was 35.06 (SD 18.36) years. The youngest participant was 1 year old while the oldest participant was aged 79 years old. The highest proportion (65/283, 22.97%) of the participants were aged between 45-54 years. Children (<15 years) made up 20.49% (58/283) of the participants while 13.07% (37/283) were more than 55 years old. Females made up a higher proportion (56.89%, 161/283) compared to males. Slightly more than half of the participants (50.88%, 144/283) were married and the majority (71.02%, 201/283) had children. Most of the participants (62.54%, 177/283) lived in rural areas and more than half of the participants had completed primary education (55.83%, 158/283).

### Personal and clinical characteristics

Less than a quarter (19.43%, 55/283) were substance abusers (used drugs, alcohol, or tobacco), less than half (44.17%, 125/283) had ever missed an ART clinic appointment, 30.04% (95/283) had ever been represented during an ART clinic appointment, 17.31 (49/283) had ever had an ART appointment representation during IAC, 16.25% (46/237) had been on ART for more than 5 years and the majority 96.11% (272/283) received ART adherence support from a caregiver. Most of the participants (74.44%, 201/283) had a suppressed viral load 6 months after the start of ART. Slightly more than half (54.06%, 153/283) had a viral load greater than 2000 copies/ml before initiation of ART, less than half (45.93%, 130/283) had a previous history of viral un-suppression before the study period, 15.55% (44/283) were underweight at the start of IAC. Most of the participants (77.74%, 220/283) had a disease stage 1 at the beginning of ART and the majority (80.57, 228/283) had never been bedridden during ART. 27.21% (77/283) had experienced at least one ART side effect while 1.14% (4/283) had experienced at least one adverse event. 5.88% (16/283) had experienced at least and 37.1% (105/283) were on the first ART regimen. Table 1 below shows the detailed characteristics of the study participants.

**Table 1:** Characteristics of the study participants

Variable	Category	Frequency (TOTAL = 283)	Percentage (TOTAL = 100%)
**Socio-demographic characteristics**			
Age in completed years	< 15	58	20.49
	15 – 24	28	9.89
	25 - 34	37	13.07
	35 – 44	65	22.97
	45 – 54	58	20.49
	55+ Years	37	13.07
Sex	Female	161	56.89
	Male	122	43.11
Marital status	Married	144	50.88
	Not Married	139	49.12
Has children	Yes	201	71.02
	No	82	29.98
Living Area	Rural	177	62.54
	Town	106	37.46
Education	Primary	158	55.83
	None	60	21.2
	Secondary	60	21.2
	University or Higher	5	1.77
**Personal characteristics**			
Lifestyle	Not a substance user	228	80.57
	Substance user	55	19.43
Ever missed an ART appointment	No	158	55.83
	Yes	125	44.17
Ever been represented on ART	No	198	69.96
	Yes	85	30.04
Represented while on IAC	No	234	82.69
	Yes	49	17.31
Duration on ART	<5 years on ART	237	83.75
	>5 years on ART	46	16.25
Caregiver support	No	11	3.89
	Yes	272	96.11
**Clinical characteristics**			
Viral load at baseline (ART start) in c/m	>1000	69	25.56
	<1000	201	74.44
Viral load before IAC in c/m	1000-2000	130	45.94
	>2000	153	54.06
Previous history of an unsuppressed viral load	No	153	54.06
	Yes	130	45.94
Normal body mass index	No	239	84.45
	Yes	44	15.55
Disease stage at ART initiation	Stage 2 or later	63	22.26
	Stage 1	220	77.74
Ever bedridden during ART	No	228	80.57
	Yes	55	19.43
Disease stage at IAC	Stage 2 or Later	59	20.85
	Stage 1	224	79.15
ART Side effects	No	206	72.79
	Yes	77	27.21
Adverse events	No	279	98.59
	Yes	4	1.41
Had an infection while on IAC	No	256	94.12
	Yes	16	5.88
Regimen	Second or higher	105	37.1
	First regiment	178	62.9

*ART *Antiretroviral Therapy, *IAC *Intensive Adherence Counselling, *C/M *Copies/ml of blood

### Barriers to ART adherence associated with viral non-suppression

In Table 2, we show the barriers to ART adherence reported by the study participants during IAC. The most frequent barriers reported were forgetfulness (58.66%, 166/283) and changes in the daily routine (45.58%, 139/283). The two least reported barriers were side effects (4.59%, 13/283) and pill burden (4.95%, 14/283). Among 3.53% (10/283) of the participants, there were no barriers to ART adherence identified during IAC.

**Table 2:** Barriers to ART adherence reported by the study participants

Nature of barrier	Disaggregation	Frequency (TOTAL = 283)	Percentage (TOTAL = 100%)
**Being away from home**	No	191	67.49
	Yes	92	32.51
**Changes in the daily routine**	No	153	54.06
	Yes	139	45.58
**Pill burden**	No	269	95.05
	Yes	14	4.95
**Feeling sick/sickness**	No	243	85.87
	Yes	40	14.13
**Clinical depression**	No	211	74.56
	Yes	72	25.44
**Forgetfulness**	No	117	41.34
	Yes	166	58.66
**Sleeping through dose times**	No	214	75.62
	Yes	69	24.38
**Drug side effects**	No	270	95.41
	Yes	13	4.59
**Distance to the health centre**	No	235	83.04
	Yes	48	16.96
**No barrier identified**	No	273	96.47
	Yes	10	3.53

#### Participants’ characteristics associated with viral suppression after IAC

In Table 3, we present the bivariate analysis of the participant characteristics and viral suppression after IAC. Statistically significant differences in viral suppression after IAC were observed among participants who; had children compared to those without (74.29% versus 25.71%, *p=0.040*), had never been represented on ART compared to those that had ever been represented (74.29% versus 25.71%, *p=0.007*), received no ART clinic appointment representation during IAC compared to those that had (87.14% versus 12.86%, *p=0.001*), had a viral load 6 months after ART start that was less than 1000 copies/ml blood compared to higher (79.80% vs 20.2%, *p<0.001*), had a viral load at IAC start that was between 1000-2000 copies/ml of blood (53.33% versus 46.67%, *p<0.001*), had a normal body weight (87.14% versus 12.86%, *p=0.034*), had no document ART-adverse events (100%, *p=0.004*), had no other infections during IAC compared to those that had (96.57% versus 3.43%, *p=0.003*) and participants that were on the first regimen compared to a second or higher regimen (67.14 versus 32.86%, *p=0.012*). See Table 3 for details.

**Table 3:** Bivariate analysis for socio-demographic and viral suppression after IAC

Variable	Category	TOTAL = 283	Suppressed after IAC		P-Value
		Frequency (Percentage)	No [Frequency (Percentage)] TOTAL = 73	Yes [Frequency (Percentage)] TOTAL = 210	
**Socio-demographic characteristics**					
Age in completed years	< 15	58 (20.49)	18 (24.66)	40 (19.05)	0.53
	15 - 24	28 (9.89)	9 (12.33)	19 (9.05)	
	25 - 34	37 (13.07)	6 (8.22)	31 (14.76)	
	35 - 44	65 (22.97)	18 (24.66)	47 (22.38)	
	45 - 54	58 (20.49)	12 (16.44)	46 (21.9)	
	55+ Years	37 (13.07)	10 (13.7)	27 (12.86)	
Sex	Female	161 (56.89)	47 (64.38)	114 (54.29)	0.133
	Male	122 (43.11)	26 (35.62)	96 (45.71)	
Marital Status	Married	144 \(50.88)	34 (46.58)	110 (52.38)	0.393
	Not Married	139 (49.12)	39 (53.42)	100 (47.62)	
Has children	Yes	201 (71.02)	45 (61.64)	156 (74.29)	0.040*
	No	82 (29.98)	28 (38.36)	54 (25.71)	
Living Area	Rural	177 (62.54)	43 (58.90)	134 (63.81)	0.456
	Town	106 (37.46)	30 (41.10)	76 (36.19)	
Education	None	60 (21.2)	15 (20.55)	45 (21.43)	0.742
	Primary	158 (55.83)	43 (58.90)	115 (54.76)	
	Secondary	60 (21.2)	13 (17.81)	47 (22.38)	
	University	5 (1.77)	2 (2.74)	3 (1.43)	
**Personal characteristics**					
Substance user±	No	159 (74.65)	36 (75.00)	123 (74.55)	0.949
	Yes	54 (25.35)	12 (25.00)	42 (25.45)	
Ever missed an ART appointment	No	158 (55.83)	37 (50.68)	121 (57.62)	0.304
	Yes	125 (44.17)	36 (49.32)	89 (42.38)	
Ever been represented on ART	No	198 (69.96)	42 (57.53)	156 (74.29)	0.007**
	Yes	85 (30.04)	31 (42.47)	54 (25.71)	
Represented while on IAC	No	234 (82.69)	51 (69.86)	183 (87.14)	0.001**
	Yes	49 (17.31)	22 (30.14)	27 (12.86)	
Duration on ART	<5 years	237 (83.75)	22 (30.14)	55 (26.19)	0.514
	>5 years	46 (16.25)	51 (69.86)	155 (73.81)	
Caregiver support	No	11 (3.89)	5 (6.85)	6 (2.86)	0.128
	Yes	272 (96.11)	68 (93.15)	204 (97.14)	
**Clinical characteristics**					
Viral load at baseline (ART start) in c/m	>1000	69 (25.56)	28 (41.79)	41 (20.20)	<0.001***
	≤1000	201 (74.44)	39 (58.21)	162 (79.80)	
Viral load before IAC in c/m	1000-2000	130 (45.94)	18 (24.66)	112 (53.33)	<0.001***
	>2000	153 (54.06)	55 (75.34)	98 (46.67)	
Previous history of a high viral load	No	153 (54.06)	22 (30.14)	131 (62.38)	<0.001***
	Yes	130 (45.94)	51 (69.86)	79 (37.62)	
Normal body mass index	No	239 (84.45)	56 (76.71)	27 (12.86)	0.034*
	Yes	44 (15.55)	17 (23.29)	183 (87.14)	
Disease stage at ART initiation	Stage 2 or Later	63 (22.26)	21 (28.77)	42 (20.00)	0.121
	Stage 1	220 (77.74)	52 (71.23)	168 (80.00)	
Ever bedridden during ART	No	228 (80.57)	56 (76.71)	172 (81.90)	0.334
	Yes	55 (19.43)	17 (23.29)	38 (18.10)	
Disease stage at IAC	Stage 2 or Later	59 (20.85)	18 (24.66)	41 (19.52)	0.352
	Stage 1	224 (79.15)	55 (75.34)	169 (80.48)	
ART Side effects	No	206 (72.79)	50 (68.49)	156 (74.29)	0.338
	Yes	77 (27.21)	23 (31.51)	54 (25.71)	
Adverse events	No	279 (98.59)	69 (94.52)	210 (100.00)	0.004**
	Yes	4 (1.41)	4 (5.48)	0 (0.00)	
Had an infection while on IAC	No	256 (94.12)	59 (86.76)	197 (96.57)	0.003**
	Yes	16 (5.88)	9 (13.24)	7 (3.43)	
Regimen	Second or higher	105 (37.1)	36 (49.32)	69 (32.86)	0.012*
	First regiment	178 (62.9)	37 (50.68)	141 (67.14)	

±Participants less than 18 years not included. Significant codes at 5%, 1% and 0.1%. *ART *Antiretroviral Therapy, *IAC* Intensive Adherence Counselling, *C/M* Copies/ml of blood

### Barriers to ART adherence associated with viral suppression after IAC

In Table 4, we show the findings of the cross-tabulation of barriers reported during IAC and viral suppression after IAC. The proportion of participants that achieved a suppressed viral load after IAC was higher among participants that had not reported any barrier related to: being away from home (66.67%, *p=0.62*), changes in the daily routine (55.71%, p=0.35), a high pill burden (96.19%, *p=0.070*), illness (86.67%, p=0.512), clinical depression (74.76%, *p=0.894*), sleeping through dosing times (76.67%, p=0.486), ART side effects (95.71%, *p=0.680*), distance (85.71%, *p=0.042*) versus to those that had reported (33.33%, 43.81%, 3.81%, 13.33%, 25.24%, 23.33%, 4.29%, and 14.29% respectively). The proportion of participants that had achieved viral suppression after IAC was higher among those that reported forgetfulness as a barrier compared to those that did not (56.19% versus 43.81%, *p=0.150*). For 3.18% (8/283) of the participants, no barriers were established during IAC.

**Table 4:** Bivariate analysis for barriers to ART adherence identified and reported during IAC and viral suppression after IAC

Nature of barrier	Disaggregation	Frequency (Percentage) TOTAL = 284	Suppressed after IAC		P-Value
			No [Frequency (Percentage)] TOTAL = 73	Yes [Frequency (Percentage)] TOTAL = 210	
**Being away from home**	No	191 (67.49)	51 (69.86)	140 (66.67)	0.616
	Yes	92 (32.51)	22 (30.14)	70 (33.33)	
**Changes in the daily routine**	No	153 (54.06)	36 (49.32)	117 (55.19)	0.310
	Yes	139 (45.58)	37 (50.68)	92 (43.81)	
**Pill burden**	No	269 (95.05)	67 (91.78)	202 (96.19)	0.070
	Yes	14 (4.95)	6 (8.22)	8 (3.81)	
**Feeling sick/sickness**	No	243 (85.87)	61 (83.56	182 (86.67)	0.512
	Yes	40 (14.13)	12 (16.44)	28 (13.33)	
**Clinical depression**	No	211 (74.56)	54 (73.97)	157 (74.76)	0.894
	Yes	72 (25.44)	19 (26.03)	53 (25.24)	
**Forgetfulness**	No	117 (41.34)	25 (34.25)	92 (43.81)	0.150
	Yes	166 (58.66)	48 (65.75)	118 (56.19)	
**Sleeping through dose times**	No	214 (75.62)	53 (72.60)	161 (76.67)	0.486
	Yes	69 (24.38)	20 (27.40)	49 (23.33)	
**Drug side effects**	No	270 (95.41)	69 (94.52)	201 (95.71)	0.680
	Yes	13 (4.59)	4 (5.48)	9 (4.29)	
**Distance to the health centre**	No	235 (83.04)	55 (75.34)	180 (85.71)	0.042*
	Yes	48 (16.96)	18 (24.66)	30 (14.29)	
**No barrier identified**	No	273 (96.47)	71 (97.26)	202 (96.19)	0.670
	Yes	10 (3.53)	2 (2.74)	8 (3.81)	

Significant code at 5%

### Multivariable log-binomial regression analysis for statistically significant variables with viral suppression

In the multivariate analysis, only factors with a p-value <0.05 at bivariate analysis were considered for the unadjusted analysis and consequently for the adjusted analysis. In the multivariable analysis, we dropped variables that did not improve the fit of the model based on the log-likelihood. In the unadjusted analysis, participants were more likely to have a suppressed viral load after IAC if they; had children (PRR 1.19, 95% CI 0.99-1.40, *p=0.063*), had a viral load 6 months after starting ART (baseline viral load) that was less than 1000 copies per ml (PRR 1.34, 95% CI 1.10-1.67, *p=0.004*), had a normal body mass index (PRR=1.25 95% CI 0.97–1.59, *p=0.007*) or if they were on the first ART regimen (PRR 1.21 95% CI 1.03-1.41, *p=0.020*). Participants were less likely to have a suppressed viral load if they: had ever sent someone to pick medication refills on their behalf during an ART clinic appointment (represented) before IAC (PRR 0.81, 95% CI0.68-0.92, *p=0.017*), represented during IAC (PRR 0.70, 95% CI 0.54-0.91, *p=0.009*), reported distance as a barrier to ART adherence (PRR 0.82, 95% CI 0.65-1.03, *p=0.084*), had a viral load at the start of IAC that was greater than 2000 copies per ml (PRR 0.74, 95% CI 0.65-0.85, *p<0.001*), had a previous history of having an unsuppressed viral load (PRR 0.71, 95% CI 0.61-0.83, *p<0.001*) or if participants had another infection during IAC (PRR 0.57, 95% CI 0.32-1.00, *p=0.048*). In the adjusted analysis, participants were less likely to achieve viral suppression after IAC if they had a viral load of more than 2000 copies/ml of blood (aPRR 0.81, 95% CI 0.70-0.93, *p=0.002*) or if participants had a previous history of having an unsuppressed viral load (aPRR 0.79, 95% CI 0.66-0.94, *p=0.007*). Having; a viral load 6 months after starting ART that was less than1000 copies per ml (aPRR 1.18, 95% CI 0.96-1.45, *p=0.109*), ever had an ART clinic appointment representation (aPPR 0.91, 95% CI 0.78-1.07, p=*0.256*), received any ART clinic representation during IAC (aPRR 0.83, 95% CI 0.65-1.06, p=*0.128*) and Being on the first ART regimen (aPRR 0.99, 95% CI 0.83-1.18, p=*0.927*) were not statistically significantly associated with viral suppression after IAC. Table 5 shows the details of the findings of the analysis.

**Table 5:** Multivariable log-binomial regression analysis results for unadjusted and adjusted prevalence risk ratios (RR) outcomes of variables with viral suppression after IAC

Variable	Category	Prevalence Risk Ratio (PRR)	P-Value	Adjusted Prevalence Risk Ratio (aPRR)	P-Value
Has children	No	1			
	Yes	1.19 (0.99 – 1.40)	0.063		
Represented for refill before IAC	No	1		1	0.256
	Yes	0.81 (0.68 - 0.92)	0.017*	0.91 (0.78 - 1.07)	
Represented for refill while on IAC	No	1		1	0.128
	Yes	0.70 (0.54 - 0.91)	0.009**	0.83 (0.65 - 1.06)	
Distance barrier identified during IAC	No	1			
	Yes	0.82 (0.65 - 1.03)	0.084		
Viral load at baseline (ART start)	>1000	1		1	0.109
	≤1000	1.34 (1.10 - 1.67)	0.004**	1.18 (0.96 - 1.45)	
Viral load at before IAC	1000-2000	1		1	0.002**
	>2000	0.74 (0.65 - 0.85)	<0.001***	0.81 (0.70 - 0.93)	
Previous history an unsuppressed viral load	No	1		1	0.007**
	Yes	0.71 (0.61 - 0.83)	<0.001***	0.79 (0.66 - 0.94)	
Normal body mass index	No	1			
	Yes	1.25 (0.97 – 1.59)	0.077		
Had an infection while on IAC	No	1			
	Yes	0.57 (0.32 - 1.00)	0.048		
Regimen	2^nd^ or higher	1			0.927
	1^st^ regimen	1.21 (1.03 - 1.41)	0.020 **	0.99 (0.83 - 1.18)	

Note significant codes at 5%, 1% and 0.1%. *ART *Antiretroviral Therapy, *IAC *Intensive Adherence Counselling, *C/M  *Copies/ml of blood

### Influencers of viral suppression after IAC

Of the three ART Clinic In-Charges interviewed for the study, two were female while one was male. The findings showed that drug availability, staffing adequacy and patient follow-up procedures influenced viral suppression after IAC. Having rare occurrences of drug shortages or procedures that enable cross-health facility drug transfers helped improve adherence and suppression. This was complemented by taking patient follow-up information and having flexible working opening hours. However, staffing shortages were highlighted as barriers that could negatively influence adherence and viral suppression. Table 6 summarizes the findings of the key informant interviews.

**Table 6:** Results of the key informant interviews

Question	Theme	*Influencers of viral suppression*
(i) Could you please describe the types of ART regimens available and the process that guides your allocation regimens to clients on ART?	Drug availability	*“We rarely have shortages of drugs because our stock is well-managed, and we always have all drugs available” -* **KYINF01**.
Describe some facility-related factors that you think affect viral suppression outcomes of patients at your family.	Staffing adequacy	*“We are missing some other cadres like dispenser, nurses, and other cadres”* – **KYINF02.** *“Yes, the clinic staffing is appropriate because we have very many health workers, we have expert clients, facility linkages all working in the ART clinic,”* ***KYINF01****.* *“Yes, we do have a trained counsellor supported by the IP,”* **KYINF03** . *“Our ART clinic is working from Monday to Friday mainly, but even over the weekends…., even if it is a weekend, we still serve them. Then for the hours, it is mainly from 8 am to 5 pm,”* **KYINF03**.
Please explain some of the procedures undertaken to ensure successful completion of IAC and how do you think these help to achieve better outcomes?	Patient follow up procedures	*“Yes, we take patient contact information,” “Especially when we are initiating them. We ask them their names, their phone numbers, where they come from. We even fill their locator forms where we even draw a map to direct us to their homes. We ask them for their commonly used names in their villages such that when they are missing, we can locate them*,” **KYINF 03**. *“Our clinic has a contact, and it is displayed on the door, so when this client comes late when the clinic is closed, they call, and we come back and serve them,”* **KYINF 01**.

## Discussion

Our study examined viral suppression among PLHIV on ART who started and completed IAC in 2019 at selected health facilities in Kamuli district, a rural district in East Central Uganda. The study findings showed that only about seven in ten PLHIV who started and completed IAC achieved viral suppression. Viral suppression after IAC was less likely among PLHIV whose viral load before IAC was >2000 copies/ml of blood compared to those that had a viral load between 1000 – 2000 copies/ml of blood. Patients with a previous history of an unsuppressed viral load we also less likely to achieve a successful viral suppression after IAC. Also, the study found that there were good measures that addressed continued drug availability, adequate staffing levels and patient follow-up procedures.

From our findings, about three in ten virally unsuppressed PLHIV did not achieve viral suppression at the end of the IAC period. The suboptimal performance could directly contribute to the country’s underachievement of the 2020 UNAIDS target (90% viral suppression among those receiving ART) [14] and the 2030 UNAIDS targets (95% viral suppression among those receiving ART) [2]. Persons with an unsuppressed viral load are more likely to transmit HIV [8, 11] and more likely to die due to the attributed causes of HIV such as AIDS [22, 23]. Interventions such as IAC are expected to achieve complete viral suppression if the ART regimen is effective [24]. The gap in suppression observed may also imply ART regimen failure [25–27] or programmatic gaps regarding the quality of IAC the degree to which this study didn’t explore further. However, the viral suppression rate observed was similar to that observed in a systematic review that reported 70% of PLHIV achieved viral suppression after implementation of adherence-targeted interventions [12, 28]. It is likely that, to improve the proportion of viral suppression, IAC may need to be coupled with other interventions. However, the findings also showed a slight improvement than that observed in 2018 in the country (64%) [10], albeit, this was observed in the general PLHIV population but not those that had been started and completed IAC. Other studies have only studied viral load suppression after IAC in children and adoloscents [29]. This study, therefore, introduces new and unique findings on the projected success of IAC in Kamuli district and possibly beyond.

The study also established that patients with higher load (>2000 copies/ml) at the start of IAC were less likely to achieve viral suppression after IAC compared to those that had a lower unsuppressed load (between 1000-2000 copies/ml). A high initial viral load is associated with a lesser likelihood of achieving viral suppression as observed by studies in Ethiopia [5], Vietnam [15] and Canada [6]. Challenges related to routine viral load monitoring may result in a delay to detect an unsuppressed viral load resulting in a higher viral load. Such challenges, though not established by this study, maybe patient-related or health-system related [30]. This finding emphasizes the need for effective and routine viral load monitoring to help the early detection of unsuppressed viral loads. Also, likely, the 6-month IAC period may not be sufficient to achieve a suppressed viral load among patients in this category. A slower rate of viral suppression is commonly observed among patients with a high viral load [31]. Therefore, patients with a higher viral count may need to be admitted into IAC longer than those with a lower viral count.

Our findings also showed that a previous history of a high viral load i.e. having a viral rebound was associated with a lower likelihood of achieving viral suppression after IAC. This finding may be attributed to multiple causes. A suboptimal regimen potency may result in an early warning of regimen failure [32, 33], unresolved barriers during IAC may also result in ART interruption during IAC [34] or observations that to a fraction of PLHIV, high viremia remains persistent [35]. A proper analysis of the situation should be done by a clinician to provide comprehensive care to this category of patients [36]. However, as noted by this study, the majority of the staff are at the ART clinics were lower cadre health care workers. In absence of a qualified health worker, patients in this category could be at risk of improper clinical-care decisions. Viral rebound could be partly attributed to poor clinical decisions on ART regimen choices for patients [32, 33].

The health facilities were routinely fully stocked with all necessary ART regimens and implied that patients never missed drugs to ART drug shortages. This may have supported the achievement of the viral suppression rate observed. The availability of different regimens free-of-charge is also associated with viral suppression as observed by studies in the United States [15], Cameroon [34] and Uganda [19]. PLHIV have free access to ART needed to achieve viral suppression. Only one health facility reported having enough health workers at the ART clinic, however, the majority of these were lower cadre workers. The dominance of lower cadre health workers without grounded clinical training in HIV care and management in addition to the low staffing levels reported by the rest of the health centres had influenced the observed viral suppression rate after IAC. Without a comprehensive analysis of the patient’s clinical and psychological status, it is easy for such to miss out on very important aspects or underlying health problems that may affect viral suppression [37].

All health facilities had a trained counsellor to support patient counselling at ART start and during IAC and all staff in the ART clinic were trained in counselling particularly IAC counselling. This was a good approach that helped to support client counselling and may have influence the viral suppression rate observed by the study [36]. Further, all health facilities offered flexible and convenient ART clinics schedules (through week and weekends). The practice of picking contact information was noticed among all health facilities and this may have influenced viral suppression as with the case observed by other studies [38].

## Study strengths and limitations

We used census data, this helped to avoid potential bias that could have occurred through using a sampling technique. The study was conducted over a short period offering the best use of the limited time and resources available for this study. The findings of this study have therefore been made available in a short period and can help IAC programming in the district and country. The study involved an examination of the most recent cohort of patients that started and completed on IAC (January 2019– December 2019). This encompassed the entire year which helps to account for any effects caused by season variations and provides the most recent information about the situation in the district. However, the retrospective nature of the study made it vulnerable to missing or incomplete information from the data sources, however, the researcher trained assistants on how to use the multiple available data sources to bridge gaps in data. Also, the researcher selected data collectors who had long-term experience in HIV-related work. The study was unable to assess the quality of IAC sessions neither was it able to collect and assess other factors that may have influenced viral suppression beyond those collected for its scope. However, the investigators reviewed various literature and included the most likely factors in the study to minimize this effect. Also, the study was conducted at only three health facilities with one district which may not be sufficient to represent the entire district or country.

## Conclusions

The viral suppression rate after IAC was lower than international and national targets threatening the achievement of the goal to end the HIV epidemic by 2030. The factors associated with viral suppression were a viral load of more than 2000 copies/ml of blood and a previous history of having an unsuppressed viral load. Virally unsuppressed patients with a viral load of more than 2000 copies/ml of blood need a closure examination during clinical practice and possibly an extended period of IAC. This may improve the viral suppression rate in this category. Further, health workers should use a previous history of an unsuppressed viral load as a marker for possible unsuccessful IAC and make more tailored follow-up interventions for patients in this category. On a positive note, there were good measures that addressed continued drug availability, adequate staffing levels and patient follow-up procedures. However, complementing the available staff cadres with the staff of higher cadres could help support improve viral suppression rates after IAC.

## Ethical consideration

Ethical approval was granted by the Clarke International University Ethical Review Board under CLARKE-2020-18. Additionally, permission and approval to conduct the study were sought and granted by the District Health Office and each of the health facility administration. Newly generated identifiers were used for the data collection, the codebook was available only to the Principal Investigator of the study to ensure the anonymity records. Additionally, the audio and transcript were anonymized not shared with any external parties to the research. To ensure confidentiality, all patient identifiers were excluded from the data and instead substituted with newly generated codes. Informed consent was obtained from all the participants before the interview. All procedures performed in the study that involved human participants were following the ethical standards of the Clarke International University Research Ethics Committee (CIUREC) and with the 1964 Helsinki declaration and its later amendments or comparable ethical standards [39]. The findings were reported following the Strengthening of the Reporting of Observational Studies in Epidemiology (STROBE) guidelines [40].

## Data Availability

The dataset used and/or analysed during the current assessment is available from the corresponding author upon reasonable request.

## Abbreviations

aPRR: Adjusted Prevalence Risk Ratio
ART: Antiretroviral therapy
EC: East Central
HC: Health centre
HIV: Human immune-deficiency virus
IAC: Intensive Adherence Counselling
MoH: Ministry of Health
PRR: Prevalence Risk Ratio
PLHIV: People living with HIV
SSS: Sub-sahara Africa
WHO: World Health Organisation
